# Assessing Upfront Treatment Patterns for Newly Initiated Patients With Pulmonary Arterial Hypertension in the United States

**DOI:** 10.36469/001c.138006

**Published:** 2025-06-05

**Authors:** Carly Paoli, Wenze Tang, Sumeet Panjabi, Ashwin Ravichandran

**Affiliations:** 1 Johnson & Johnson, Titusville, New Jersey, USA; 2 Ascension St Vincent Heart Center, Indianapolis, Indiana, USA

**Keywords:** real-world study, treatment patterns, combination therapy, dual therapy, guidelines, pulmonary arterial hypertension

## Abstract

**Background:** The 2022 European Society of Cardiology/European Respiratory Society (ESC/ERS) pulmonary hypertension guidelines recommend initial combination of endothelin receptor antagonist (ERA) and phosphodiesterase type-5 inhibitor (PDE5i) in patients with pulmonary arterial hypertension (PAH) at low to intermediate risk without cardiopulmonary comorbidities. **Objective:** To examine US treatment patterns for newly diagnosed patients, including frequency of cardiopulmonary comorbidities. **Methods:** Treatment-naïve adults (≥18 years) initiating treatment, identified using claims data (IQVIA PharMetrics® Plus; April 2013–June 2023), were assigned dual therapy if initiating ERA/PDE5i within a treatment-determination period (3 months), or monotherapy if initiating ERA or PDE5i. Descriptive statistics captured 25th/75th percentiles, means (SD), and medians. **Results:** Of 2868 patients, 824 (28.7%) initiated dual therapy and 2044 (71.3%) monotherapy. In dual therapy, 461 (56.0%) initiated ERA first, 250 (30.3%) PDE5i first, and 113 (13.7%) both the same day. In monotherapy, 153 (7.5%) received ERA and 1891 (92.5%) PDE5i. For escalation to dual therapy, 330 (16.1%) monotherapy users initiated ERA (10.7%) or PDE5i (5.5%) during follow-up. Most had cardiopulmonary comorbidities (monotherapy: 86.8%; dual: 79.6%). Of the 824 on dual therapy, 20.4% started triple therapy during follow-up. Compared with monotherapy, dual therapy users were younger (54.9 vs 59.6 years) and mostly female (72.9% vs 60.9%). **Discussion:** This study found that in the United States, among newly diagnosed PAH patients, 71.3% initiated monotherapy and 28.7% dual therapy, with 16.1% of monotherapy patients eventually escalating to dual therapy. High rates of initial monotherapy may reflect the high proportion of patients with comorbidities and their possible intolerance of initial dual therapy. As these data mostly precede the 2022 guidelines, future research should include treatment post-guidelines, rationales behind decision making, differences between initial monotherapy and dual therapy users, and monotherapy overreliance and effects on morbidity and mortality. **Conclusions:** This analysis of real-world US treatment patterns for newly initiating PAH patients found low rates of upfront dual-therapy use with high rates of cardiopulmonary comorbidities.

## INTRODUCTION

Pulmonary arterial hypertension (PAH) is characterized by progressively increasing pulmonary vascular resistance, leading to right ventricular failure and death.^1^ Despite considerable progress in new treatment strategies for PAH over the past 2 decades, mortality remains high.^2^ Current guidance for the management of patients with PAH, from the 2022 European Society of Cardiology/European Respiratory Society (ESC/ERS) guidelines for the diagnosis and treatment of pulmonary hypertension (PH), recommends regular multiparametric risk assessment to determine patients’ risk of deterioration and death, with the aim of implementing a treatment strategy that prevents disease progression and achieves low-risk status.^2^ These guidelines build on initial recommendations for combination or rapid sequential therapy outlined in the 2015 guidelines.^3^ For patients at low or intermediate risk without cardiopulmonary comorbidities, the recommendation is initial dual therapy with an endothelin receptor antagonist (ERA) and a phosphodiesterase type-5 inhibitor (PDE5i). For patients with cardiopulmonary comorbidities, evidence-based treatment decisions are challenging because of scarce clinical trial data.^2^ For these patients, the ESC/ERS guidelines recommend initial monotherapy with either a PDE5i or an ERA, with further treatment decisions to be made on an individual basis.^2^ The 2019 American College of Chest Physicians Guideline and Expert Panel Report recommends initial dual therapy for patients with World Health Organization functional class (FC) II and patients with FC III without disease progression or poor prognosis; for patients with FC III and rapid disease progression or poor prognosis and for patients with FC IV, initial parenteral prostanoid therapy is recommended.^4^ However, these guidelines have not been updated for some years.

Increasing attention is being given to real-world studies examining the implementation of guideline-recommended care.^5,6^ However, little has been published on contemporary, real-world treatment patterns in PAH, specifically on the use of initial combination therapy. There is insufficient real-world evidence characterizing patients treated with initial ERA and PDE5i dual therapy. The aim of this retrospective cohort study was to examine real-world data on the extent of initial dual-therapy utilization and associated time to receipt of therapy, and to compare the clinical characteristics of patients selected for initial dual therapy with the characteristics of those chosen for initial monotherapy. Considering the recommendation for initial ERA or PDE5i monotherapy among patients with cardiopulmonary comorbidities, we assessed the frequency of these comorbidities among initial monotherapy and initial dual therapy cohorts to gauge if and how they may impact the implementation of PAH therapeutics. Additionally, patient characteristics that are potentially predictive of initial dual therapy were identified and assessed.

## METHODS

### Data Source

The study used the IQVIA PharMetrics® Plus commercial insurance claims database, which provides a diverse representation of employers, payers, providers, and geographic zones covering 50 US states. The database contains historical information on patient demographics, plan enrollment, and claims for inpatient, outpatient, and pharmacy treatment (with associated costs) for approximately 60 million patients. The population enrolled in IQVIA PharMetrics® Plus is generally representative, with respect to age and sex, of the commercially insured US population aged under 65 years. Data were de-identified as per the Health Insurance Portability and Accountability Act. No written informed consent or institutional review board approval was required. This study followed the Strengthening the Reporting of Observational Studies in Epidemiology (STROBE) reporting guidelines.^7^

### Patient Eligibility Criteria

Patients were eligible for the study if they were at least 18 years of age, had at least 1 diagnosis code for PH (*Internal Classification of Diseases* [ICD], *Ninth Revision*, 416.0 primary PH and 416.8 PH secondary/unspecified; and ICD, *Tenth Revision*, I27.0 primary PH, I27.20 PH secondary/unspecified, I27.21 secondary PAH, and I27.89 other specified pulmonary heart diseases) in an inpatient or emergency room setting and at least 2 diagnoses of PH on separate days in an outpatient setting, had a new ERA or PDE5i claim, and were continuously enrolled with medical and pharmacy benefits during the 6-month baseline period before the index date. To avoid inclusion of patients receiving a PDE5i for erectile dysfunction, those initiating a PDE5i at index were required to have a prescription for at least 2 pills per day (for PAH, sildenafil is taken ≥3 times per day and tadalafil is taken ≥2 times per day or once daily in the form of 2 pills [total 40 mg]). Patients were excluded if they had received a PAH medication during a 6-month window prior to their first observed PAH therapy (baseline).

### Study Design

**Figure 1.**
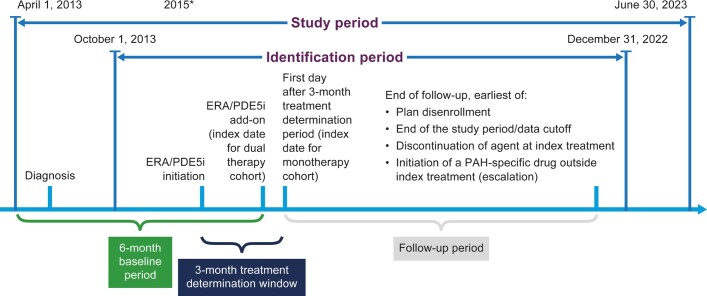
Study Design Abbreviations: ERA, endothelin receptor antagonist; PDE5i, phosphodiesterase type-5 inhibitor. *Time point when upfront dual therapy became the standard of care.

The study design is shown in **Figure 1**. Patients initiating an ERA or a PDE5i between October 1, 2013, and December 31, 2022, were identified; the full study period was April 1, 2013, to June 30, 2023, to allow for a 6-month baseline period. Assignment to the dual-therapy or monotherapy cohort was based on a 3-month treatment determination period that began at initiation of the first ERA or PDE5i. This 3-month period reflects the authorization and processing time that it can take in real-world US clinical practice for patients to obtain ERA medication even when the ERA and the PDE5i are prescribed at the same time for use as dual therapy.

The index date was the date of initiation of the second drug for the dual-therapy cohort, and the first day after the 3-month treatment determination period for the monotherapy cohort. The baseline period was the 6 months prior to the index date. Patients were followed until plan disenrollment, study end date (June 30, 2023), discontinuation of an agent in their index treatment, or initiation of any PAH-specific drugs outside their index treatment (escalation).

Patients were assigned to the dual-therapy cohort if they initiated both an ERA and a PDE5i within the 3-month treatment determination period, regardless of the order of initiation. Patients were assigned to the monotherapy cohort if they initiated either an ERA or a PDE5i within the 3-month period. The index date was the date of initiation of the second drug for the dual-therapy cohort, and the first day after the 3-month treatment determination period for the monotherapy cohort. The baseline period was the 6 months prior to the index date. Patients were followed until plan disenrollment, study end date (June 30, 2023), discontinuation of an agent in their index treatment, or initiation of any PAH-specific drugs outside their index treatment (escalation).

### Study Variables and Analysis

Patient characteristics included demographics, calendar year of initial therapy use, baseline comorbidities and Quan-Charlson Comorbidity Index^8^ (**Table S1**), and PAH symptoms during baseline. Descriptive statistics were conducted with counts, 25th and 75th percentiles, means (SD), and medians (range). The standardized mean difference (SMD) between the monotherapy and dual-therapy groups was calculated for patient characteristics, with SMD >0.1 considered to indicate a meaningful between-group difference.^9^ The monotherapy and dual-therapy cohorts were not propensity matched.

As the main analyses for the initial monotherapy and dual-therapy cohorts were determined using a 3-month window, we described the time from diagnosis to first medication start, treatment duration, reasons for therapy discontinuation, and patient characteristics. Patients were followed up until data cutoff (June 30, 2023) or plan disenrollment. Patients were considered as discontinuing their PAH therapy if the gap in prescription refill (of any PAH medication in the therapy) was over 3 months. Treatment duration was summarized using Kaplan-Meier statistics (medians and interquartile range [IQR]).

LASSO^10–12^ regression (an interpretation-friendly statistical learning procedure) was also conducted to identify the most important patient characteristics predictive of dual therapy. Input features included demographics (sex, age, US census region, insurance type, health plan type), etiologies (HIV, connective tissue disease, portal hypertension, congenital heart disease), comorbidities (cardiopulmonary [hypertension, diabetes, coronary artery disease, obesity] and others [congestive heart failure, atrial fibrillation, chronic obstructive pulmonary disease, depression]), PAH-related symptoms (syncope, peripheral edema, malaise/fatigue, dyspnea, hemoptysis, chest pain, dizziness, abnormal gait, cardiomegaly, ascites), and concomitant medications (corticosteroids, diuretics, opioids). A 10-fold cross validation was used to select the best model, which was then refitted using all data. Analyses were conducted using R (version 4.0.2).

## RESULTS

### Patients and Patient Characteristics

**Figure 2.**
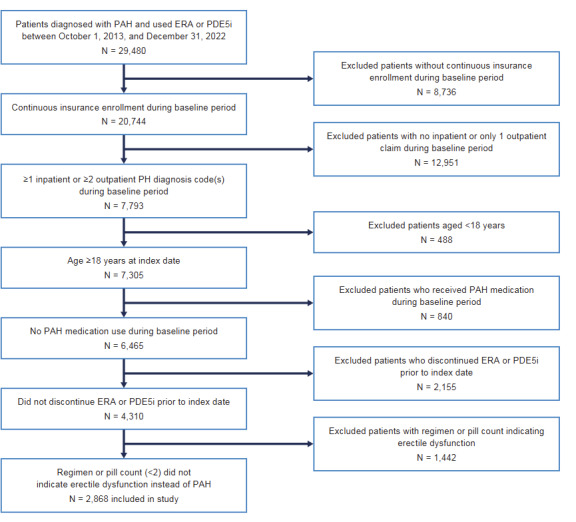
Cohort Attrition Flow Abbreviations: ERA, endothelin receptor antagonist; PAH, pulmonary arterial hypertension; PDE5i, phosphodiesterase type-5 inhibitor.

The total number of patients diagnosed with PAH and receiving an ERA or a PDE5i during the identification period was 29 480. After applying inclusion and exclusion criteria, 2868 patients were included in the analysis (**Figure 2**). Patients receiving dual therapy were younger than those receiving monotherapy (mean age, 54.9 vs 59.6 years; SMD = 0.345), and, for the most part, the proportion of patients receiving monotherapy increased with increasing age (8.2%, 35-44 years; 17.2%, 45-54 years; 36.0%, 55-64 years; 32.9%, ≥65 years [SMD = 0.329]) (**Table 1**). There was a higher proportion of women in the dual-therapy vs monotherapy group (72.9% vs 60.9%; SMD = 0.258) (**Table 1**). Uptake of dual therapy rose over time, with 24.5% of patients initiating dual therapy in 2013-2016 compared with 33.4% in 2017-2019 and 31.0% in 2020-2022 (SMD = 0.367) (**Table 1**). Both cohorts had a similar duration of follow-up: 16.7 months in the dual-therapy cohort and 15.2 months in the monotherapy cohort (SMD = 0.062). While there was a significant difference between the utilization of combination therapy over time vs monotherapy, there was a slight decrease in this difference during the COVID-19 years (2020-2022).

**Table 1.** Patient Baseline Demographic Characteristics Using the 3-Month Treatment Determination Period to Define Dual Therapy

**Characteristic**	**Initial Monotherapy**	**Initial Dual Therapy**	**SMD^a^**
N	2044	824	
Age at baseline, mean years (SD)	59.6 (14.3)	54.9 (13.1)	0.345
Age categories at baseline, n (%)			0.329
18-24 years	30 (1.5)	12 (1.5)	
25-34 years	86 (4.2)	50 (6.1)	
35-44 years	167 (8.2)	116 (14.1)	
45-54 years	352 (17.2)	182 (22.1)	
55-64 years	736 (36.0)	293 (35.6)	
≥65 years	673 (32.9)	171 (20.8)	
Male sex, n (%)	799 (39.1)	223 (27.1)	0.258
US regions, n (%)			0.084
East	310 (15.2)	136 (16.5)	
Midwest	533 (26.1)	187 (22.7)	
South	779 (38.1)	332 (40.3)	
West	422 (20.6)	169 (20.5)	
Healthcare plan, n (%)			0.069
HMO	527 (25.8)	224 (27.2)	
Other	153 (7.5)	74 (9.0)	
PPO	1364 (66.7)	526 (63.8)	
Commercial insurance plan, n (%)	1011 (49.5)	366 (44.4)	0.101
Period of therapy initiation, n (row %)			0.367
2013-2016 (n = 1286)	971 (75.5)	315 (24.5)	
2017-2019 (n = 773)	515 (66.6)	258 (33.4)	
2020-2022 (n = 809)	558 (69.0)	251 (31.0)	
Follow-up duration, median (IQR) (mo)	15.2 (5.8-30.6)	16.7 (5.8-34.4)	0.062
On-treatment duration, Kaplan-Meier median (IQR) (mo)	14.0 (3.4-48.6)	13.9 (4.4-31.2)	

Abbreviations: HMO, health maintenance organization; IQR, interquartile range; PPO, preferred provider organization; SMD, standardized mean difference.

The SMD between the monotherapy and dual therapy groups was calculated for patient characteristics; an SMD >0.1 was considered as having a meaningful between-group difference.

Most patients in the study had cardiopulmonary comorbidities (diabetes, hypertension, obesity, coronary artery disease): 86.8% in the monotherapy and 79.6% in the dual-therapy cohort (**Table 2**). In total, 541 (26.5%) patients receiving initial monotherapy and 191 (23.2%) patients receiving initial dual therapy had at least 3 cardiopulmonary comorbidities (**Table 2**). More patients in the monotherapy cohort had PAH associated with congenital heart disease (11.2% vs 4.5%; SMD = 0.252) and more patients in the dual-therapy cohort had PAH associated with connective tissue disease (27.3% vs 17.2%; SMD = 0.246) (**Table 2**).

**Table 2.** Patient Baseline Comorbidities, PAH Etiology, and Concomitant Medication Using the 3-Month Treatment Determination Period to Define Dual Therapy

**Characteristic**	**Initial Monotherapy**	**Initial Dual Therapy**	**SMD^a^**
N	2044	824	
Any cardiopulmonary issues (diabetes, hypertension, obesity, coronary artery disease), n (%)	1775 (86.8)	656 (79.6)	0.194
Comorbidities, n (%)			
Diabetes without complications	714 (34.9)	209 (25.4)	0.21
Diabetes with complications	308 (15.1)	90 (10.9)	0.124
Obesity	648 (31.7)	288 (35.0)	0.069
Congestive heart failure	1360 (66.5)	512 (62.1)	0.092
Atrial fibrillation	562 (27.5)	88 (10.7)	0.438
Coronary artery disease	591 (28.9)	188 (22.8)	0.14
COPD	814 (39.8)	277 (33.6)	0.129
Hypertension	362 (17.7)	127 (15.4)	0.062
No. of cardiopulmonary comorbidities, n (%)			0.21
0	269 (13.2)	168 (20.4)	
1	611 (29.9)	249 (30.2)	
2	623 (30.5)	216 (26.2)	
3	413 (20.2)	152 (18.4)	
4	128 (6.3)	39 (4.7)	
QCCI, mean (SD)	3.66 (2.43)	3.57 (2.59)	0.037
QCCI categories, n (%)			0.163
Low (0)	85 (4.2)	54 (6.6)	
Mild (1, 2)	548 (26.8)	256 (31.1)	
Moderate (3, 4)	850 (41.6)	293 (35.6)	
Severe (≥5)	561 (27.4)	221 (26.8)	
PAH etiology, n (%)			
Connective tissue disease	351 (17.2)	225 (27.3)	0.246
HIV	29 (1.4)	17 (2.1)	0.049
Portal hypertension	106 (5.2)	63 (7.6)	0.101
Congenital heart disease	229 (11.2)	37 (4.5)	0.252
Methamphetamine substance use disorder	17 (0.8)	18 (2.2)	0.111
PCH	9 (0.4)	9 (1.1)	0.075
PVOD	256 (12.5)	114 (13.8)	0.039
Concomitant medications, n (%)			
Anti-anxiety agents	400 (19.6)	149 (18.1)	0.038
Anti-arrhythmic agents	371 (18.2)	125 (15.2)	0.080
Antibiotics	1214 (59.4)	406 (49.3)	0.204
Antidepressants	699 (34.2)	208 (25.2)	0.197
Antidiabetic agents	596 (29.2)	167 (20.3)	0.207
Antihyperlipidemics	909 (44.5)	256 (31.1)	0.279
Antihypertensives	967 (47.3)	327 (39.7)	0.154
Antiplatelet agents	180 (8.8)	48 (5.8)	0.115
Antineoplastic agents	144 (7.0)	47 (5.7)	0.055
Anti-inflammatory agents	301 (14.7)	142 (17.2)	0.068
Beta blockers	1039 (50.8)	315 (38.2)	0.256
Calcium channel blockers	603 (29.5)	263 (31.9)	0.052
Cardiotonic agents	254 (12.4)	73 (8.9)	0.116
Corticosteroids	818 (40.0)	310 (37.6)	0.049
Diuretics	1586 (77.6)	591 (71.7)	0.135
Opioids	1091 (53.4)	408 (49.5)	0.077

Abbreviations: COPD, chronic pulmonary obstructive disease; HIV, human immunodeficiency virus; PAH, pulmonary arterial hypertension; PCH, pulmonary capillary hemangiomatosis; PVOD, pulmonary veno-occlusive disease; QCCI, Quan-Charlson Comorbidity Index; SMD, standardized mean difference.

^a^The SMD between the monotherapy and dual-therapy groups was calculated for patient characteristics; an SMD >0.1 was considered as having a meaningful between-group difference.

### Treatment Patterns

In all, 2044 (71.3%) patients received initial monotherapy and 824 (28.7%) received initial dual therapy, defined during the 3-month treatment determination period. Of patients receiving dual therapy, 461 (56.0%) initiated an ERA first, 250 (30.3%) initiated a PDE5i first, and 113 (13.7%) initiated both agents on the same day. Of the monotherapy patients, 153 (7.5%) received an ERA and 1891 (92.5%) a PDE5i. The time from first PAH diagnosis to first medication start was shorter for patients receiving dual therapy compared with those receiving monotherapy (median, 4.3 months [IQR 2.0-10.9] vs 6.7 months [IQR 4.3-14.6], respectively). In total, 330 (16.1%) initial monotherapy users were escalated to dual therapy with the addition of an ERA (10.7%) or a PDE5i (5.5%) during follow-up; another 10 (0.5%) initiated a soluble guanylate cyclase stimulator during follow-up. Among the 824 initial dual-therapy users during follow-up, 20.4% were escalated to triple therapy with the addition of a prostacyclin pathway agent and 1.2% were escalated to triple therapy with the addition of a soluble guanylate cyclase stimulator. The proportion of patients receiving dual therapy at any time increased to 41.2% over the course of the entire follow-up period (**Table 3**).

**Table 3.** Treatment Patterns of Patients Receiving Dual Therapy at Any Time During the Follow-up Period

	**No. (%) of Patients**	**Time to Escalation from Monotherapy to Dual Therapy (Days)**		**No. (%) of Patients Initiating Dual Therapy Within Specified Time Window**		
		**Mean (SD)**	**Median (25th, 75th Percentiles)**	**≤30 Days**	**≤91 Days**	**≤182 Days**
Initiated ERA + PDE5i at any point during follow-up	1182	105.5 (181.3)	35 (8, 120)	560 (47.4)	824 (69.7)	985 (83.3)
ERA only → ERA + PDE5i	584 (49.4)	88.0 (162.6)	31.5 (13, 80)	289 (49.5)	461 (78.9)	515 (88.2)
Ambrisentan → ambrisentan + sildenafil	142 (12.0)	92.2 (160.4)	42 (18, 79)	61 (43.0)	116 (81.7)	124 (87.3)
Ambrisentan → ambrisentan + tadalafil	127 (10.7)	64.4 (129.4)	16 (4, 45)	84 (66.1)	107 (84.3)	115 (90.6)
Bosentan → bosentan + sildenafil	13 (1.1)	76.7 (61.3)	78 (8, 113)	5 (38.5)	7 (53.8)	13 (100)
Bosentan → bosentan + tadalafil	4 (0.3)	121.0 (97.7)	96.5 (62.5, 179.5)	0	1 (25.0)	3 (75.0)
Macitentan → macitentan + sildenafil	187 (15.8)	88.0 (155.3)	35 (18, 78)	85 (45.5)	150 (80.2)	165 (88.2)
Macitentan → macitentan + tadalafil	111 (9.4)	110.0 (212.9)	34 (12, 112)	54 (48.6)	80 (72.1)	95 (85.6)
PDE5i only → ERA + PDE5i	485 (41.0)	151.4 (208.0)	84 (20, 196)	158 (32.6)	250 (51.5)	357 (73.6)
Sildenafil → sildenafil + ambrisentan	107 (9.1)	182.9 (211.4)	120 (36, 253)	23 (21.5)	45 (42.1)	73 (68.2)
Sildenafil → sildenafil + bosentan	11 (0.9)	98.1 (85.6)	85 (14, 131)	3 (27.3)	6 (54.5)	9 (81.8)
Sildenafil → sildenafil + macitentan	140 (11.8)	174.6 (223.0)	111 (34, 215.5)	33 (23.6)	60 (42.9)	95 (67.9)
Tadalafil → tadalafil + ambrisentan	140 (11.8)	102.5 (191.9)	26 (7, 118)	78 (55.7)	101 (72.1)	116 (82.9)
Tadalafil → tadalafil + bosentan	3 (0.3)	127.0 (93.9)	165 (20, 196)	1 (33.3)	1 (33.3)	2 (66.7)
Tadalafil → tadalafil + macitentan	84 (7.1)	161.8 (206.1)	101.5 (32, 198)	20 (23.8)	37 (44.0)	62 (73.8)
ERA + PDE5i (on same day)	113 (9.6)			113 (100)	113 (100)	113 (100)
Sildenafil + ambrisentan	18 (1.5)			18 (100)	18 (100)	18 (100)
Sildenafil + bosentan	1 (0.1)			1 (100)	1 (100)	1 (100)
Sildenafil + macitentan	10 (0.8)			10 (100)	10 (100)	10 (100)
Tadalafil + ambrisentan	64 (5.4)			64 (100)	64 (100)	64 (100)
Tadalafil + bosentan	3 (0.3)			3 (100)	3 (100)	3 (100)
Tadalafil + macitentan	17 (1.4)			17 (100)	17 (100)	17 (100)

Abbreviations: ERA, endothelin receptor antagonist; PDE5i, phosphodiesterase type-5 inhibitor.

The reasons for study discontinuation are shown in **Table S2**. A total of 227 (27.6%) of initial dual-therapy users discontinued a PDE5i first, and 158 (19.2%) discontinued an ERA first. The median Kaplan-Meier on-treatment duration of initial dual therapy was 14.0 months (IQR, 3.4-48.6). In comparison, the median Kaplan-Meier on-treatment duration for the initial monotherapy users was 13.9 months (IQR, 4.4-31.2), with 420 (20.5%) patients censored for escalating to a second PAH therapy from the classes of PDE5i, ERA, or prostacyclin pathway agent.

Based on the LASSO regression to identify the most important patient characteristics predictive of dual-therapy use, the overall prediction accuracy per area under the curve of the best model fit was 0.721 (out of 1). The top 5 predictors indicating higher likelihood of initial dual-therapy use were dyspnea, cardiomegaly, connective tissue disease, portal hypertension, and use of a calcium channel blocker (**Table 4**). The 5 negative predictors indicating the least likelihood of initial dual therapy were atrial fibrillation, congenital heart disease, age at baseline, use of antibiotics, and use of antidepressants.

**Table 4.** Identified Patient Characteristics Predictive of Dual Therapy

**Characteristic**	**Coefficient**	**Odds Ratio**
Dyspnea	0.27	1.31
Cardiomegaly	0.21	1.24
Connective tissue disease	0.15	1.16
Portal hypertension	0.07	1.08
Use of calcium channel blockers	0.04	1.04
Chest pain	0.03	1.04
Syncope	0.03	1.03
HIV	0.02	1.02
Malaise/fatigue	-0.02	0.98
Peripheral edema	-0.03	0.97
Use of antidiabetic agents	-0.03	0.97
Use of antineoplastic agents	-0.03	0.97
Health plan PPO	-0.03	0.97
Cardiopulmonary comorbidity	-0.04	0.96
Use of beta blockers	-0.05	0.95
US region (Midwest)	-0.05	0.95
Abnormal gait	-0.08	0.92
Use of antihyperlipidemics	-0.08	0.92
Male sex	-0.16	0.85
Use of antidepressants	-0.17	0.85
Use of antibiotics	-0.18	0.84
Age at baseline	-0.20	0.82
Congenital heart disease	-0.20	0.82
Atrial fibrillation	-0.30	0.74

Abbreviation: PPO, preferred provider organization.

## DISCUSSION

Giving patients access to guideline-recommended care is essential to preserving their lives, especially when the recommendations are based on the risk of 1-year mortality in a rare, progressive, fatal disease like PAH. Thus, examining real-world treatment patterns is the first step in understanding the current state of how guideline-recommended care is being implemented. Our study found that only 28.7% of US patients with PAH are started on initial dual therapy. Over the course of the follow-up period, the proportion receiving dual therapy at any point rose to 41.2%, similar to previously diagnosed patients in the Registry to Evaluate Early and Long-term PAH Disease Management (46%), which finished enrolling in 2009.^13^ Patients starting on an ERA had a shorter time to escalation than those who started on a PDE5i. From 2013-2016 to 2017-2019, prescribing of initial dual therapy rose, possibly reflecting implementation of the ESC/ERS 2015 guidelines.^3^ However, the increase in prescription of dual therapy stalled during the COVID-19 period (2020-2022), as seen in a medical chart review of patients with PAH in several European countries, as well as a US-based claims analysis^14,15^; during this time, diagnosis and treatment rates were lowest in the second half of 2020 and first half of 2021.

In our study, fewer patients initiating dual therapy had comorbidities compared with those initiating monotherapy. However, most patients had cardiopulmonary comorbidities (86.8% on monotherapy; 79.6% on dual therapy). This finding does not concur with current ESC/ERS treatment guidelines for PH, which recommend that patients with cardiopulmonary comorbidities are treated initially with either an ERA or a PDE5i.^2^ The aim of this cautious treatment approach is to prevent a comorbid condition becoming worse.^2^ Our data suggest clinicians might favor starting patients on dual therapy once it has been decided that PAH is the predominant pathophysiology, regardless of any comorbidities, as suggested by the treatment algorithm task force at the 7th World Symposium on Pulmonary Hypertension.^16^

Increasing attention is being paid to real-world studies that provide data to complement clinical trials.^5,6^ A 2021 German study of newly diagnosed patients with PAH showed that 71% initiated treatment with monotherapy, 23% with dual therapy, and 6% with triple therapy,^17^ which is similar to the proportions in our study. This German study also found that patients who were older (>65 years) and had cardiovascular comorbidities or risk factors were more likely to receive monotherapy than dual therapy.^17^ Another German real-world study found that the reasons for prescribing monotherapy differed between younger (≤68 years) and older (>68 years) patients, with a diagnosis of mild PAH given as the main reason in younger patients and the presence of comorbidities as the main explanation in older patients.^18^

A large European registry-based study showed that although combination therapy increased between 2010 and 2019, the majority of patients (46.5%) still received initial monotherapy 3 years after a diagnosis of PAH.^19^ A US-based administrative claims study conducted between 2010 and 2015 showed that 94% of patients with newly diagnosed PAH initiated treatment with monotherapy, with 17% switching to combination therapy during the study.^20^ In a 2022 US-based real-world study by Pizzicato et al, a 30-day window was used to determine initial combination therapy use; this resulted in 12.2% of initial dual therapy with an ERA and a PDE5i and 9.0% of another combination therapy (triple, with a soluble guanylate cyclase stimulator).^21^ In contrast, in our study, dual-therapy use with an ERA and a PDE5i was higher (28.7%), likely due to the longer time period (3 months) used to define combination therapy. Our 3-month window may be a more realistic time frame due to the prior authorization delays that can be impacted in shorter determination periods.

The results from our study and those described above^17–21^ indicate that most patients with PAH receive initial monotherapy. Our study and both studies from Germany^17,18^ also suggest a consistent association of prescription of monotherapy with older age and the presence of comorbidities. In particular, from our LASSO regression, our study showed that atrial fibrillation, congenital heart disease, age, use of antibiotics, and use of antidepressants were associated with the least likelihood of initial dual therapy; dyspnea, cardiomegaly, connective tissue disease, portal hypertension, and use of a calcium channel blocker had the greatest likelihood of initial dual therapy.

The low rates of initial dual-therapy treatment practices in the real world are discouraging given the outcomes that dual therapy can provide as demonstrated in randomized, double-blind trials, such as AMBITION, SERAPHIN, TRITON, and ADUE. These studies demonstrated improved clinical outcomes in patients receiving initial dual therapy with an ERA and a PDE5i compared with those receiving monotherapy.^22–27^ Future research is needed to understand the reasons for the lack of upfront dual-therapy use in patients initiating PAH therapy.

### Limitations

The limitations of our study are common to studies that use retrospective claims data. In particular, administrative claims data are subject to potential coding errors and inconsistencies, data cannot be generalized to the entire US population, the presence of a claim for a dispensed prescription does not indicate that the medication was taken as prescribed, PAH medications or other medications that were administered in the inpatient setting are not captured in the database, and information such as clinical and laboratory results and disease-specific parameters such as risk score are not readily recorded in claims data. Accurate identification of patients with PAH is a challenge in analyses of claims data because no specific diagnostic code for PAH exists (forthcoming in ICD-11). Identification of patients in our study relied on a diagnostic code for PH and prescription of an ERA or a PDE5i (with restriction of PDE5 inhibitors to PAH-specific doses and regimens). Right heart catheterization was not an inclusion criterion as a previous study has validated the identification algorithm used in our study (and other similar PAH studies) and has shown that including an right heart catheterization requirement does not increase the sensitivity or positive predictive value of the algorithm.^28^

Nevertheless, claims data are extremely valuable for evaluation and characterization of treatment patterns because they represent paid pharmacy claims, as in our study.

## CONCLUSIONS

The high rate of initial monotherapy could be a result of the high proportion of patients with cardiopulmonary comorbidities in our study. Dissemination and ongoing uptake of the 2022 ESC/ERS guideline recommendations are likely to change future treatment patterns. Further education is needed to help the medical community understand and incorporate the guidelines, and to ensure patients are being treated at PH centers with demonstrated competencies in the evaluation and treatment of PAH. Future research should evaluate how to tailor and optimize treatment strategies for patients with comorbidities to narrow the treatment gap between patients with and without comorbidities.

### Declarations

C.J.P. and W.T. are employees and stockholders of Johnson & Johnson, Titusville, New Jersey. S.P. was an employee of Johnson & Johnson, Titusville, New Jersey, at the time this study was conducted. A.R. reports speakers’ bureau participation for United Therapeutics and Johnson & Johnson.

## Supplementary Material

Online Supplementary Material

## Data Availability

The data that support the findings of this study are available on request from IQVIA PharMetrics® Plus. The data are not publicly available due to privacy or ethical restrictions.
