# Supplementary material for: Assessing Upfront Treatment Patterns for Newly Initiated Patients With Pulmonary Arterial Hypertension in the United States
Source: J Health Econ Outcomes Res. 2025 Jun 5;12(1):213–21. doi: 10.36469/001c.138006 (PMC12145179; doi:10.36469/001c.138006)
Supplement: Online Supplementary Material [file jheor_2025_12_1_138006_287775.pdf]

Journal of Health Economics and Outcomes Research

Online Supplementary Material

Assessing Upfront Treatment Patterns for Newly Initiated Patients with Pulmonary Arterial Hypertension in the United States. *JHEOR*. 2025;12(1):213-221. doi:10.36469/jheor.2025.138006

Table S1: Quan-Charlson Comorbidity Index

Table S2: Reasons for Study Discontinuation

This supplementary material has been provided by the authors to give readers additional information about their work.

This is an open-access article distributed under the terms of the Creative Commons Attribution 4.0 International License (CCBY-4.0). View this license's legal deed at http://creativecommons.org/licenses/by/4.0 and legal code at http://creativecommons.org/licenses/by/4.0/legalcode for more information.

Table S1. Quan-Charlson Comorbidity Index

Comorbidities	ICD-9-CM Codes	ICD-10-CM Codes	Quan 2011 Weight
Myocardial infarction	410.%, 412.%	I21.%, I22.%, I25.2%	0
Congestive heart failure	398.91, 402.01, 402.11, 402.91, 404.01, 404.03, 404.11, 404.13, 404.91, 404.93, 425.4%-425.9%, 428.%	I09.9%, I11.0%, I13.0%, I13.2%, I25.5%, I42.0%, I42.5%-I42.9%, I43.%, I50.%, P29.0%	2
Peripheral vascular disease	093.0%, 437.3%, 440.%, 441.%, 443.1%-443.9%, 447.1%, 557.1%, 557.9%	I70.%, I71.%, I73.1%, I73.8%, I73.9%, I77.1%, I79.0%, I79.2%, K55.1%, K55.8%, K55.9%, V43.4%, Z95.8%, Z95.9%	0
Cerebrovascular disease	362.34, 430.%-438.%	G45.%, G46.%, H34.0%, I60.%-I69.%	0
Dementia	290.%, 294.1%, 331.2%	F00.%-F03.%, F05.1, G30.%, G31.1%	2
Chronic pulmonary disease	416.8%, 416.9%, 490.%-505.%, 506.4%, 508.1%, 508.8%	I27.8%, I27.9%, J40.%-J47.%, J60.%-J67.%, J68.4%, J70.1%, J70.3%	1
Connective tissue/rheumatic disease	446.5%, 710.0%-710.4%, 714.0%-714.2%, 714.8%, 725.%	M05.%, M06.%, M31.5%, M32.%-M34.%, M35.1%, M35.3%, M36.0%	1
Peptic ulcer disease	531.%-534.%	K25.%-K28.%	0
Mild liver disease	070.22, 070.23, 070.32, 070.33, 070.44, 070.54, 070.6%, 070.9%, 570.%, 571.%, 573.3%, 573.4%, 573.8%, 573.9%	B18.%, K70.0%-K70.3%, K70.9%, K71.3%-K71.5%, K71.7%, K73.%, K74.%, K76.0%, K76.2%-K76.4%, K76.8%, K76.9%, V42.7%, Z94.4%	2
Diabetes without chronic complications/mild to moderate	250.0%-250.3%; 250.8%, 250.9%	E10.0%, E10.1%, E10.6%, E10.8%, E10.9%, E11.0%, E11.1%, E11.6%, E11.8%, E11.9%, E12.0%, E12.1%, E12.6%, E12.8%, E12.9%, E13.0%, E13.1%, E13.6%, E13.8%, E13.9%, E14.0%, E14.1%, E14.6%, E14.8%, E14.9%	0
Paraplegia and hemiplegia	334.1%, 342.%, 343.%, 344.0%-344.6%, 344.9%	G04.1%, G11.4%, G80.1%, G80.2%, G81.%, G82.%, G83.0%-G83.4%, G83.9%	2
Renal disease	403.01, 403.11, 403.91, 404.02, 404.03, 404.12, 404.13, 404.92, 404.93, 582.0%-583.7%, 585.%, 586.%, 588.0%	I12.0%, I13.1%, N03.2%-N03.7%, N05.2%-N05.7%, N18.%, N19.%, N25.0%, V42.0%, V45.1%, V56.%, Z49.0%-Z49.2%, Z94.0%, Z99.2%	1
Diabetes with chronic complications	250.4%-250.7%	E10.2%-E10.5%, E10.7%, E11.2%-E11.5%, E11.7%, E12.2%-E12.5%, E12.7%, E13.2%-E13.5%, E13.7%, E14.2%-E14.5%, E14.7%	1
Any malignancy, including lymphoma and leukemia, except malignant neoplasm of skin	140.%-172.%, 174.%-195.8%, 200.%-208.%, 238.6%	C00.%-C26.%, C30.%-C34.%, C37.%-C41.%, C43.%, C45.%-C58.%, C60.%-C76.%, C81.%-C85.%, C88.%, C90.%-C97.%	2
Moderate or severe liver disease	456.0%-456.2%, 572.2%-572.8%	I85.0%, I85.9%, I86.4%, I98.2%, K70.4%, K71.1%, K72.1%, K72.9%, K76.5%, K76.6%, K76.7%	4
Metastatic solid tumor	196.%-199.%	C77.%-C80.%	6
AIDS/HIV	042.%, 043.%, 044.%	B20.%-B22.%, B24.%	4

The table above outlines the updated QCI index (Quan H, et al., *Am J Epidemiol.* 2011;173(6):676-682.). To quantify comorbidity, the QCI score is computed by adding the weights that are assigned to the specific diagnoses. Each diagnosis is only counted once. The minimum possible score is 0 and the maximum possible score is 24. The algorithms for defining comorbidities in ICD-9-CM and ICD-10-CM administrative data were coded using the approach described in Quan H, et al. *Med Care.* 2005;43(11):1130-1139.

Table S2. Reasons for Study Discontinuation

	Initial Monotherapy (N = 2044)	Initial Dual Therapy (N = 824)
Data cutoff or plan disenrollment	643 (31.5)	261 (31.7)
Discontinuation of ERA	25 (1.2)	158 (19.2)
Discontinuation of PDE5i	956 (46.8)	227 (27.6)
Initiation of other PAH medication	420 (20.6)	178 (21.6)

Abbreviations: ERA, endothelin receptor antagonist; PAH, pulmonary arterial hypertension; PDE5i, phosphodiesterase type 5 inhibitor
All values are presented as n (%).
